# Glans tunnel‐based reconstruction with skin flap or lip mucosa graft for distal penile urethral strictures

**DOI:** 10.1002/bco2.70097

**Published:** 2025-11-11

**Authors:** Fuhao Ji, Lin Wang, Tong Zhao, Yidong Liu, Xiangguo Lyu

**Affiliations:** ^1^ Department of Urology, Renji Hospital, School of Medicine Shanghai Jiao Tong University Shanghai China; ^2^ Department of Urology Jiaozuo People's Hospital Jiaozuo China

**Keywords:** glans penis, mouth mucosa, pedicled flaps, urethral stricture

## Abstract

**Purpose:**

The aim of this study is to evaluate the clinical efficacy of glans tunnel urethroplasty using a pedicled penile flap or lower lip mucosa for glans penis urethral stricture.

**Methods:**

A retrospective analysis was conducted on 41 patients with urethral stricture at the glans penis who visited our hospital from January 2021 to July 2025. Causes include iatrogenic factors (including prostate hyperplasia surgery, transurethral resection of bladder lesions and catheterisation), a history of hypospadias repair and penile lichen sclerosus (LS), including 14 cases of urethral meatus stricture and 27 cases of navicular fossa stricture. The length of the stricture was 2.0 (1.0, 3.0) cm, the maximum flow rate (*Q*_max_) was (7.24 ± 3.47) ml/s, the International Prostate Symptom Score (IPSS) was (17.49 ± 5.29), and the International Index of Erectile Function ‐ 5 (IIEF‐5) was 14.0 (9.00, 21.00) points. Surgery preserved glans integrity, incised the ventral stenotic urethra and reconstructed the defect with a flap or mucosa graft. A F16/18 indwelling catheter was removed after 3 weeks. Pre‐ and post‐operative uroflowmetry, IPSS and IIEF‐5 evaluations were conducted.

**Results:**

All post‐operative outcomes were assessed at 12–23 months (median 20, IQR 19–21). Postoperative *Q*_max_ increased to 23.61 ± 4.54 ml/s, and the IPSS decreased to 9.02 ± 3.57 (both *P* < 0.001 vs. pre‐op). The IIEF‐5 score (14.71 ± 6.48) points showed no significant change. No recurrence, glans dehiscence, fistula, infection or ischaemia occurred.

**Conclusions:**

Glans tunnel urethroplasty maintaining glans integrity effectively treats urethral meatal and navicular fossa strictures, balancing function and aesthetics.

## 1 INTRODUCTION

Urethral stricture is a prevalent urological condition, with an estimated incidence ranging from 229 to 627 cases per 100 000 individuals.^1^ It constitutes approximately 0.6% of the high‐risk population. Some types are extremely complex and difficult to repair,^2^ but anterior urethral strictures are more common, accounting for 92.2% of all urethral strictures,^3^ and among these, fossa navicularis strictures represent 18%.^4^ The primary aetiologies of meatal and fossa navicularis strictures include iatrogenic factors, such as transurethral resection of the prostate (TURP) and catheterisation, a history of hypospadias repair, and penile lichen sclerosus (LS). Clinically, patients typically exhibit voiding dysfunction, manifesting as decreased urinary stream force, spraying and elevated post‐void residual urine volume. With the increasing utilisation of transurethral procedures, the incidence of meatal and fossa navicularis strictures has risen. Nevertheless, no consensus exists on the optimal treatment approach. Repairing these strictures poses a dual challenge: restoring unobstructed micturition while preserving the aesthetic integrity of the glans penis and reconstructing a near‐normal slit‐like urethral meatus.^5^

Current treatment options for urethral meatus and fossa navicularis strictures include simple dilation, meatotomy and urethroplasty. The American Urological Association (AUA) guidelines recommend that for simple male meatal or fossa navicularis strictures, initial treatment can be either urethral dilation or meatotomy; otherwise, early urethroplasty should be considered as the primary intervention.^6^ Various surgical techniques have been described for treating meatal stenosis and fossa navicularis strictures, including meatotomy, techniques preserving the glans penis, the use of flaps or free grafts, and both ventral or dorsal penile approaches to widen the urethra and perform anastomotic urethroplasty.^7^, ^8^, ^9^, ^10^, ^11^, ^12^ Surgical complications primarily include glans dehiscence, urethral diverticulum and recurrence of the stricture, with techniques preserving the glans being advantageous.^5^, ^9^, ^10^, ^11^ Additionally, the most commonly used tissues for urethral reconstruction include penile skin flaps and oral mucosa grafts (OMG).^13^, ^14^, ^15^, ^16^ For patients with insufficient or unsuitable preputial tissue because of post‐circumcision, multiple surgeries, or LS, penile skin flaps are not appropriate, making oral mucosa grafts the preferred option, with variations in the selection of lip mucosa, buccal mucosa and lingual mucosa.

This study investigates the application of a glans‐preserving approach, specifically the glans tunnel pedicled penile skin flap urethroplasty. This technique avoids incisions in the coronal sulcus and glans. Depending on the patient's individual characteristics, either a penile pedicled flap or lower lip mucosa is selected as the material for urethral repair. The post‐operative outcomes of these patients, including urinary flow rates (*Q*_max_), International Prostate Symptom Score (IPSS), and results from the International Index of Erectile Function ‐ 5 (IIEF‐5) questionnaire, are comprehensively described.

## 2 PATIENTS AND METHODS

### 2.1 Patients and surgical technique

A retrospective analysis was conducted on meatal and fossa navicularis stricture patients treated at our institution from January 2021 to July 2025. The follow‐up lasted at least 12 months. The median age at surgery was 61.5 ± 14.1 years. Approved by the ethics committee, the study included pre‐operative clinical data collection, physical examination, urine culture and cystoscopy. Post‐operatively, prophylactic antibiotics were given for 3 days, with further treatment based on urinalysis results. Routine pre‐ and post‐operative evaluations covered urine flow rate, IPSS and IIEF‐5.

**FIGURE 1:**
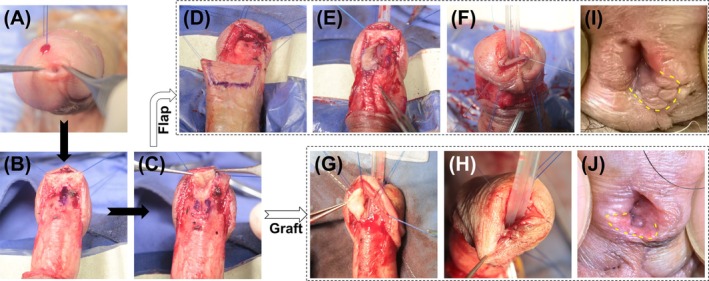
Illustration of the surgical procedure. (A) The stenotic external urethral orifice; (B) identification and marking of the length of the urethral stricture segment; (C) longitudinal incision of the stricture segment; (D and G) harvesting of the pedicled flap or oral mucosa; (E, F and H). traction of the pedicled flap or oral mucosa through the glans tunnel to the urethral meatus; (J and K) appearance 1 year after the operation for urethral stricture (the yellow dotted line indicates the transferred flap and graft).

The patient underwent surgery in a supine position under general intravenous anaesthesia. Thirty minutes before surgery, first or second‐generation cephalosporins were administered. After routine disinfection and draping, a circular incision was made 0.5 cm from the coronal sulcus and carried down through Buck's fascia until the corpus spongiosum was exposed. Dissection was carried out to the dorsal side of the urethral sponge and along the glans penis. The urethra was dissected retrogradely to the meatus, forming a tunnel for the glans while preserving the integrity of the coronal sulcus and glans. The length of the stricture was marked (Figure 1B). A vertical incision was made on the ventral side of the stricture, and dissection continued proximally until the normal urethral mucosa was exposed (Figure 1C). A urethral probe was used to confirm proximal urethral patency. The length of the stricture was measured, and appropriate urethral mucosal length was obtained. Based on the patient's prepuce condition and aetiology, either a pedicled flap or oral mucosa was chosen (Figure 1D, G). This study used pedicled flaps including ventral, dorsal or lateral penile skin flaps and oral mucosa, typically lower lip mucosa, as grafts. The above‐mentioned tissues were retracted with two sutures and pulled through the glans tunnel to the urethral meatus, with a F16/18 silicone catheter placed (Figure 1E–H). The flap or graft was sutured intermittently to the cut edges of the urethral sponge using 6–0 sutures. The wound was closed in layers, disinfected again, covered with gauze and compressed with an elastic bandage.

### 2.2 Statistical analysis

Using Statistical Package for the Social Sciences (SPSS) 25 statistical software, Shapiro–Wilk normality test was used to test normality. Normally distributed continuous data are expressed as mean ± standard deviation (x̄ ± s). For continuous variables that do not follow a normal distribution, data are expressed as median (Q1, Q3). Paired *t*‐tests were employed for between‐group comparisons when the differences followed a normal distribution. A *p*‐value of <0.05 is considered statistically significant.

## 3 RESULTS

In the analysis involving 41 patients, the aetiologies were as follows: 26 cases resulted from transurethral surgery, eight from catheter‐related factors, and seven from penile LS. Specifically, 14 patients had meatal stenosis, while 27 had fossa navicularis stricture. They were divided into the Flap Group (*n* = 28) and the Oral Mucosa Graft Group (*n* = 13) according to different repair materials, among which all seven cases of LS were repaired with oral mucosa (Table 1). The mean length of the urethral stricture measured 2.0 (1.0, 3.0) cm. Pre‐operatively, the maximum urinary flow rate (*Q*_max_) was (7.24 ± 3.47) ml/s, the IPSS was (17.49 ± 5.29), and the IIEF‐5 score was 14.0 (9.00, 21.00) points.

**TABLE 1:** Comparison between the flap group and the oral mucosa graft group.

	Flap group (*n* = 28)	Oral mucosa graft group (*n* = 13)
LS cases	0 cases	7 cases (53.8%)
Non‐LS aetiologies	Iatrogenic(22 cases) Catheter related (six cases)	Iatrogenic (four cases) catheter‐related (two cases)
1‐year post‐operative Recurrence rate (LS patients)	Not included	0%

All postoperative outcomes were assessed with a follow‐up period ranging from 12 to 23 months, with a median duration of 20 months and an interquartile range (IQR) of 19–21 months. The catheter was removed 3 weeks post‐surgery, and all patients regained normal urination. Postoperatively, *Q*_max_ increased to (23.61 ± 4.54) ml/s, and IPSS decreased to (9.02 ± 3.57), showing statistically significant differences compared to preoperative values (*P* < 0.001). However, the postoperative IIEF‐5 score of 14.71 ± 6.48 points did not differ significantly from the pre‐operative score.

**FIGURE 2:**
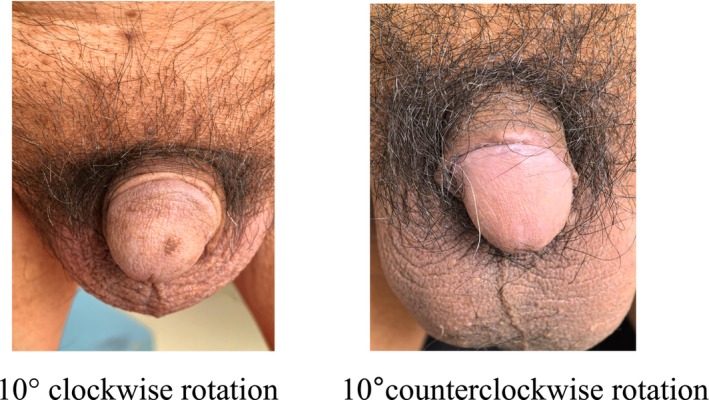
A small subset of patients exhibited penile torsion, which, however, did not impair urination, sexual function, or cause psychological disturbances. 10° clockwise rotation, 10°counterclockwise rotation.

At 30 days post‐external meatus stricture surgery, the sutures were noted to gradually detach, and the transferred pedicled skin flap recovered its normal colour, showing no signs of necrosis. Thirty days following fossa navicularis stricture surgery, the urethral mucosa appeared pink, indicating the absence of stricture. Throughout the post‐operative follow‐up period, no meatal stenosis or stricture recurrence was detected (Figure 1J, K). Additionally, no urinary fistula, wound infection or ischaemic necrosis cases were observed. The patients were satisfied with both the urinary function and the aesthetic appearance of the meatus. Within 3 months after catheter removal, 13 patients experienced urinary stream splitting or spraying. One year later, four patients still reported the continuation of these symptoms. Seven cases (17.1%) had mild penile torsion, manifested as a rotation of less than 10° in the penile shaft during erection, which did not significantly affect the symmetry of the overall appearance; the remaining 34 cases (82.9%) had no obvious torsion in the erect state, and the penile axis remained naturally straight (Figure 2).

## 4 DISCUSSION

In urological reconstructive surgery, treating meatal and fossa navicularis strictures requires balancing function and aesthetics. In this glans tunnel technique urethroplasty, our post‐operative follow‐up 12–23 months (median 20, IQR 19–21) showed no recurrence of urethral stricture or urinary fistula, indicating a high success rate. Additionally, the aesthetic outcome was favourable, leading to high patient satisfaction. Our study suggests that this glans‐preserving approach is an effective clinical technique.

The rising use of endoscopic procedures like cystoscopy and ureteroscopy has increased the annual incidence of meatal and fossa navicularis strictures, likely because of penile compression and ischaemia.^7^ Repairing penile urethral strictures requires restoring urethral function and meeting glans aesthetic standards. Traditional conservative treatments, such as urethral dilation and self‐catheterisation, have a high 2‐year recurrence rate (up to 85%) and can cause secondary long‐segment strictures.^6^, ^17^ A ventral incision for urethral fistula repair improves patency but risks glans appearance and function. In contrast, advanced urethral repair techniques, with an 87% success rate, better balance function restoration and aesthetic preservation.^6^

Urethral repair techniques have evolved greatly. Early procedures mainly used ventral longitudinal incisions at the urethral meatus and fossa navicularis. Cohney^18^ and Blandy's^19^ flap methods for fossa navicularis strictures failed because of complications like penile skin deformities. Jordan^20^ improved the approach with a ventral transverse island flap, achieving good results in five patients (median 17‐month follow‐up). Early surgeries split the glans for direct repair, but this risked glans splitting, emphasising the need to preserve glans integrity.

Glans‐sparing urethroplasty was a major breakthrough. Armenakas and McAninch's^10^ ‘glans‐cap’ technique via a coronal incision reduced scarring and splitting risks, with only one recurrence in 15 patients over 42 months. De Laet^11^ combined buccal mucosa grafting and dorsal mucosal covering for 10 meatal and fossa navicularis stricture cases, reporting no glans splitting or fistulas after 1 year, ideal for severe defects. DANESHVAR^5^ used buccal mucosa grafts for incision‐free repair of urethral meatus and navicular fossa strictures. Transurethrally implanted after resection, this approach benefited three patients. However, insufficient space exposure is an issue, and large‐scale studies are needed to determine its application scope and complication rate. Overall, glans‐sparing repair is a proven approach.

In clinical practice of urethral stricture repair, patient‐specific conditions must be considered to weigh the benefits and drawbacks of different tissue repair materials to select the most suitable and effective repair plan. Currently, with advances in autologous tissue transfer technology, commonly used materials include pedicled flaps and free mucosa such as oral mucosa, bladder mucosa and intestinal mucosa. Penile skin flaps, widely used for urethral repair, are hairless, easy to harvest and handle, compatible in a moist environment, and have good blood supply and extensibility. Despite some narrowing in long penile skin flap harvesting, the length of the stricture is often short, preventing changes in penile appearance. In our patients with meatal and fossa navicularis strictures, the pedicled skin flap can be from the ventral, dorsal or lateral aspects without excessive dissection of the dartos fascia, ensuring adequate mobility and preventing penile torsion. For patients with insufficient preputial tissue because of post‐circumcision, multiple surgeries, or LS, oral mucosal grafts are the preferred option.^13^ Lip mucosa, buccal mucosa and tongue mucosa have similar excellent tissue properties. The procedure is straightforward and can be performed by a single physician. According to our post‐operative follow‐up results, lip mucosal grafts achieve good repair outcomes, and patients can resume eating on the first post‐operative day. Notably, because the graft is taken from a superficial area, haemostasis with gelatine sponge is satisfactory, eliminating the need for sutures, thus preventing any deformity of the lower lip. However, if there is post‐operative bleeding, the use of silver ion dressings and gauze with pressure bandages provides effective haemostasis.

Considering the compression of the cavernosum of the penis, in order to avoid postoperative stenosis, the intraoperative urethral meatus forming, when the flap or graft is anastomosed with the urethra, it is necessary to form an ‘ectropion’ state as far as possible, to achieve over‐correction, to leave room for post‐operative contracture, and to avoid the newly formed urethral meatus contracting and narrowing again. This technique involves carefully dissecting the stricture segment and preserving the glans and coronal sulcus integrity, leading to a natural and aesthetically pleasing penile appearance. The intact glans structure provides solid support for the new urethra, which may help reduce post‐operative diverticula and urinary fistula.^21^ The glans tunnel urethroplasty for meatal and fossa navicularis strictures aligns with penile anatomy and provides patient satisfaction with urination and appearance. Because stricture length is positively correlated with severe cavernous fibrosis, resulting in poorer outcomes, and the glans tunnel technique requires a smaller flap, it is suitable even for patients without phimosis or after circumcision, without increasing the risk of donor site complications. Neighbouring flaps are easily transferable and highly reliable.

The key to success in this technique includes (1) sufficient tunnelling space, (2) preservation of flap blood supply and overcorrection of contraction to mitigate inevitable contraction, (3) the urethral scar was fully excised to reduce fibrosis and provide a stable new urethra, and (4) preservation of the complete glans anatomy to reduce diverticula and avoid urine leakage. This technique shows good results in urination and does not negatively affect sexual function. Similar glans‐preserving techniques using oral mucosa flaps also achieve good results.^22^, ^23^, ^24^ Nevertheless, if the stricture is because of LS, combining oral mucosa with the tunnel may prevent disease progression.^25^ For patients who refuse urethral repair surgery, paclitaxel‐coated balloons (PCBs) can be used as a preferred minimally invasive alternative. However, whether different anatomical locations are suitable for anterior urethral strictures still requires further research.^26^

Current limitations of this study include its single‐centre retrospective design, relatively small patient sample and short follow‐up duration. Additionally, there is no consensus on the definition of urethroplasty success, and post‐operative assessment methods are limited. Therefore, long‐term follow‐up is essential to clarify surgical efficacy.

## CONFLICT OF INTEREST STATEMENT

The authors have no conflicts of interest to disclose.

## References

[1] Santucci RA , Joyce GF , Wise M . Male urethral stricture disease. J Urol. 2007;177(5):1667–1674. 10.1016/j.juro.2007.01.041 17437780

[2] Wang L , Song W , Peng X , Lyu R , Wang J , Jin C , et al. Redo inferior pubectomy for failed anastomotic urethroplasty in pelvic fracture urethral injury. Curr Urol. 2024;18(1):30–33. 10.1097/CU9.0000000000000224 38505155 PMC10946640

[3] Abdeen BM , Leslie SW , Badreldin AM . Urethral strictures. [Updated 2024 Oct 29]. In: StatPearls [Internet]. Treasure Island (FL): StatPearls Publishing; 2025 Available from: https://www.ncbi.nlm.nih.gov/books/NBK564297/ 33231967

[4] Fenton AS , Morey AF , Aviles R , Garcia CR . Anterior urethral strictures: etiology and characteristics. Urology. 2005;65(6):1055–1058. 10.1016/j.urology.2004.12.018 15913734

[5] Daneshvar M , Hughes M , Nikolavsky D . Surgical management of fossa navicularis and distal urethral strictures. Curr Urol Rep. 2018;19(6):43. 10.1007/s11934-018-0792-1 29667080

[6] Wessells H , Morey A , Souter L , Rahimi L , Vanni A . Urethral stricture disease guideline amendment (2023). J Urol. 2023;210(1):64–71. 10.1097/JU.0000000000003482 37096574

[7] Meeks JJ , Barbagli G , Mehdiratta N , Granieri MA , Gonzalez CM . Distal urethroplasty for isolated fossa navicularis and meatal strictures. BJU Int. 2012;109(4):616–619. 10.1111/j.1464-410X.2011.10248.x 21615852

[8] Morey AF , Lin HC , DeRosa CA , Griffith BC . Fossa navicularis reconstruction: impact of stricture length on outcomes and assessment of extended meatotomy (first stage Johanson) maneuver. J Urol. 2007;177(1):184–187. 10.1016/j.juro.2006.08.062 17162036

[9] Chowdhury PS , Nayak P , Mallick S , Gurumurthy S , David D , Mossadeq A . Single stage ventral onlay buccal mucosal graft urethroplasty for navicular fossa strictures. Indian J Urol. 2014;30(1):17–22. 10.4103/0970-1591.124200 24497676 PMC3897046

[10] Armenakas NA , Morey AF , McAninch JW . Reconstruction of resistant strictures of the fossa navicularis and meatus. J Urol. 1998;160(2):359–363. 10.1016/S0022-5347(01)62895-7 9679877

[11] De Laet C , De Win G . Glans preserving buccal mucosa urethroplasty for glandular and distal urethral strictures. Turk J Urol. 2022;48(4):309–314. 10.5152/tud.2022.22024 35913448 PMC9612693

[12] Barbagli G , Montorsi F , Guazzoni G , Larcher A , Fossati N , Sansalone S , et al. Ventral oral mucosal onlay graft urethroplasty in nontraumatic bulbar urethral strictures: surgical technique and multivariable analysis of results in 214 patients. Eur Urol. 2013;64(3):440–447. 10.1016/j.eururo.2013.05.046 23759258

[13] Sjöqvist S , Ishikawa T , Shimura D , Kasai Y , Imafuku A , Bou‐Ghannam S , et al. Exosomes derived from clinical‐grade oral mucosal epithelial cell sheets promote wound healing. J Extracell Vesicles. 2019;8(1):1565264. 10.1080/20013078.2019.1565264 30719240 PMC6346716

[14] Bryk DJ , Yamaguchi Y , Zhao LC . Tissue transfer techniques in reconstructive urology. Korean J Urol. 2015;56(7):478–486. 10.4111/kju.2015.56.7.478 26175866 PMC4500804

[15] Snodgrass W . Tubularized, incised plate urethroplasty for distal hypospadias. J Urol. 1994;151(2):464–465. 10.1016/s0022-5347(17)34991-1 8283561

[16] Hmida W , Othmen MB , Bako A , Jaidane M , Mosbah F . Penile skin flap: a versatile substitute for anterior urethral stricture. Int Braz J Urol. 2019;45(5):1057–1063. 10.1590/S1677-5538.IBJU.2018.0652 31038860 PMC6844360

[17] Veeratterapillay R , Pickard RS . Long‐term effect of urethral dilatation and internal urethrotomy for urethral strictures. Curr Opin Urol. 2012;22(6):467–473. 10.1097/MOU.0b013e32835621a2 22773058

[18] Cohney BC . A penile flap procedure for the relief of meatal stricture. Br J Urol. 1963;35(2):182–183. 10.1111/j.1464-410x.1963.tb02615.x 14022148

[19] Blandy JP , Tresidder GC . Meatoplasty. Br J Urol. 1967;39(5):633–634. 10.1111/j.1464-410x.1967.tb11804.x 6073105

[20] Jordan GH . Reconstruction of the fossa navicularis. J Urol. 1987;138(1):102–104. 10.1016/s0022-5347(17)43006-0 3599186

[21] Özbey H . The mystery of Jacob Henle's ‘septum glandis’. J Anat. 2019;234(5):728–729. 10.1111/joa.12965 30861113 PMC6481410

[22] Zumstein V , Dahlem R , Maurer V , Marks P , Kluth LA , Rosenbaum CM , et al. Single‐stage buccal mucosal graft urethroplasty for meatal stenoses and fossa navicularis strictures: a monocentric outcome analysis and literature review on alternative treatment options. World J Urol. 2020;38(10):2609–2620. 10.1007/s00345-019-03035-8 31786639

[23] Wirtz M , Claeys W , Francois P , Waterloos M , Waterschoot M , Lumen N . Treatment of meatal strictures by dorsal inlay oral mucosa graft urethroplasty: a single‐center experience. J Clin Med. 2021;10(19):4312. Published 2021 Sep 22. 10.3390/jcm10194312 34640331 PMC8509526

[24] Farrell MR , Campbell JG , Zhang L , Nowicki S , Vanni AJ . Transurethral reconstruction of fossa navicularis strictures with dorsal inlay buccal mucosa graft urethroplasty. World J Urol. 2022;40(6):1523–1528. 10.1007/s00345-022-03994-5 35384486

[25] Tausch TJ , Peterson AC . Early aggressive treatment of lichen sclerosus may prevent disease progression. J Urol. 2012;187(6):2101–2105. 10.1016/j.juro.2012.01.071 22503028

[26] Kapriniotis K , Loufopoulos I , Apostolopoulou A , Anderson PCB , Papaefstathiou E . Drug‐coated balloon treatment for urethral strictures: is this the future? A review of the current literature. J Clin Med. 2025;14(8):2854. 10.3390/jcm14082854 40283682 PMC12027672

